# Plant Phenomics: Emerging Transdisciplinary Science

**DOI:** 10.34133/2019/2765120

**Published:** 2019-01-22

**Authors:** Seishi Ninomiya, Frédéric Baret, Zong-Ming (Max) Cheng

**Affiliations:** ^1^University of Tokyo, Tokyo, Japan; ^2^French National Institute of Agricultural Research, Paris, France; ^3^Nanjing Agricultural University, Nanjing, China; ^4^University of Tennessee, TN, USA

 Humankind is facing an unprecedented challenge to produce enough food for the coming decades because of population growth and increase in the average demand per capita, changes in climate conditions, and limitations in arable land area, as well as pressure on the water and resources. Two main avenues should be concurrently taken to increase crop productivity: improving genetics to get more efficient and resilient crops and developing optimal crop management practices. The description and understanding of crop functioning will therefore be instrumental both for genetic improvement and crop management. It will help to associate functional traits with the genome which will accelerate genetic progress by having more efficient techniques to design ideotypes adapted to particular pedoclimatic and crop management conditions and create them from the available genetic diversity. Similarly, knowledge of plant functioning will provide ways to take strategic and tactical decisions for optimal crop management within a given pedoclimatic, technical, and socioeconomic context. The modeling of crop functioning appears thus as a key element to formalize the accumulated knowledge on the ecophysiological processes that drive plant growth under given environmental conditions. Such models have already been developed for several species. They are based on the description of elementary processes using either mechanistic or empirically based approaches. These models are assembled at the plant and canopy levels to account for the complexity of the interactions between them. The validation and calibration of such models require conducting and compiling a large range of experiments under contrasted environmental conditions. However, such experiments targeting the elementary processes or the functioning of the whole plant and canopy need an ensemble of complementary measurements that are generally expensive, destructive, and low-throughput. While such detailed ecophysiological measurements are expected to be achieved, they can be complemented by repeated observations of the form and structure of organs, plants, canopies, and cellular components from which information on the corresponding functioning will be extracted. This corresponds to the emerging domain of plant phenomics.

The plant phenome is defined as the plant characteristics resulting from the realization of the genetic program stored in the cell under given environmental conditions. The targeted characteristics are either structural (dimensions, shape, position, and orientation of the cells, organs, and plants), biochemical, or based on energy or mass fluxes. The dynamics of the measured quantities is of prime importance to access more directly the underlying ecophysiological processes that determine the plant phenome. Plant phenomics can therefore be defined as the science of plant phenome characterization. It should be also extended to the description of the organ, plant, or canopy functioning.

Plant phenomics has been rapidly emerging as an independent research field in parallel to the technological advances in sensors, vectors, communication, and geolocalization systems and in signal and image processing and the associated computation capacity, as well as in all other omics tools. A brief bibliometric study shows that the number of publications related to plant phenomics per year has been rapidly growing since 2010 (Figure 1). The development of plant phenomics was mostly boosted by breeders for genetic improvements to match the maturity reached by the high-throughput genotyping techniques. As a matter fact, the value of the detailed and complete description of plant genomes can only be maximized if functions or traits are associated with the genes. Large efforts have therefore been dedicated to the development of high-throughput phenotyping techniques to break this main bottleneck of genetic improvement. In parallel, the community is also rapidly growing alongside the creation of national, continental, and international plant phenotyping infrastructures and research centers. These centers and research networks facilitate the exchange of ideas, results, data, and codes to create the necessary standards and to train generations of scientists in plant phenomics.

Plant phenomics is a transdisciplinary domain encompassing physics, biology, genetics, statistics, computer science, metrology, and other related disciplines. Such an extended academic sphere creates a challenge for the growing plant phenomics community to establish a proper publishing platform for sharing new results from the research activities up to their targeted applications, presenting reviews on specific aspects of plant phenotyping, and discussing positions on new developments. This is currently done either within general crop-science journals or through journals specialized in a single discipline having some connection with plant phenotyping. The rapid advance and growth of the plant phenomics discipline call for a dedicated journal in plant phenomics, integrating with computing science, engineering science, and multibiological sciences. Launching* Plant Phenomics* became possible through the support of Nanjing Agricultural University, which is building the world-class of Plant Phenomics Center, and through a partnership with the American Association for the Advancement of Science, publisher of* Science Magazine*.

The intended coverage of* Plant Phenomics *can be broken down into five areas (Figure 2):

1. ***High-throughput data acquisition*** for cells, organs, individual plants, and plant canopy including both the root system and the aerial parts of all plant species, under controlled or field conditions. It includes the related technological advances about sensors, vehicles, robotics, calibration, and metrology issues.
2. ***Data management*** including information systems, ontology, metainformation, data mining, data sharing, and standardization.
3. ***Data interpretation*** to transform raw measurements into usable traits. It covers computer vision, signal processing, machine learning, and statistical and physically based approaches.
4. ***Modeling*** the plant structure and its dynamics and plant functioning through detailed description of ecophysiological processes. This also includes sensitivity analyses, model calibration and validation, and data assimilation.
5. ***Integration and applications*** including integration with other omics and other disciplines for advancing plant genetics, breeding, precision agriculture, and supporting decision systems at the field and farm levels, as well as resource management at the larger scales.

*Plant Phenomics *will be an open-access, online-only journal, allowing scientific discoveries to be quickly and freely disseminated to all interested viewers. In addition to research articles, the journal will also welcome news and views on hot topics and current events and provoking opinions with broad interest to the plant phenomics community.

We are honored to serve as the founding Editors-in-Chief (EiC) and are so grateful to have an exceptional group of highly qualified, energetic associate editors (AEs) across the world to help us in building* Plant Phenomics* into a leading journal for the plant phenomics research community by attracting high level papers and becoming an integral reference in the field.

*Seishi Ninomiya* *Frédéric Baret* *Zong-Ming (Max) Cheng*

## Figures and Tables

**Figure 1:**
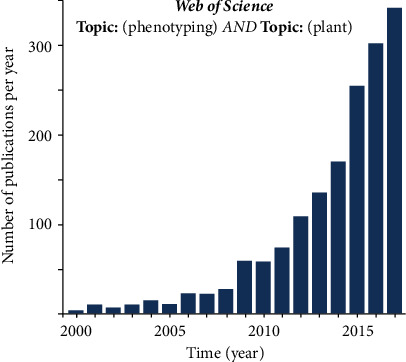
Number of publications per year related to plant phenomics.

**Figure 2:**
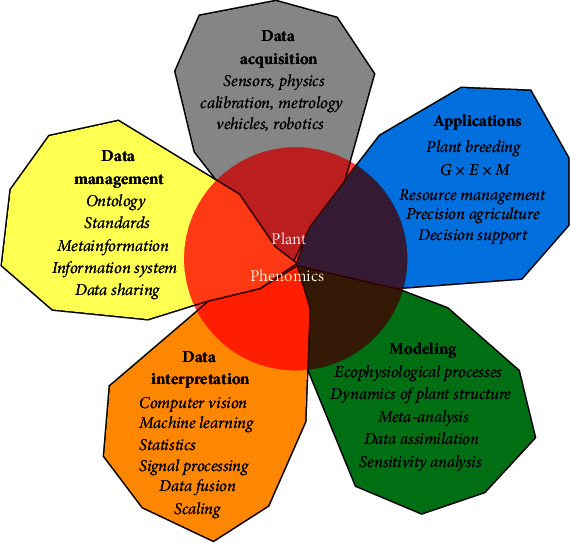
The five pillars of plant phenomics.

